# Engagement of CD300c by a Novel Monoclonal Antibody Ameliorates Behavioral Deficits in a 5xFAD Mouse Model of Alzheimer’s Disease

**DOI:** 10.3390/biomedicines13051169

**Published:** 2025-05-10

**Authors:** Suin Lee, Chang Ki Lim, Jongyeob Kim, Joon Kim, Hee Kyung Jin, Jae-sung Bae, Jae-Won Jeon

**Affiliations:** 1 CentricsBio, Inc., 3F, BK tower, 28, Beobwon-ro 11-gil, Songpa-gu, Seoul 05836, Republic of Korea; 2 Department of Laboratory Animal Medicine, College of Veterinary Medicine, Kyungpook National University, Daegu 37224, Republic of Korea; 3 Department of Physiology, School of Medicine, Kyungpook National University, Daegu 37224, Republic of Korea

**Keywords:** Alzheimer’s disease, monocyte-derived macrophage, macrophage differentiation, CD300c, 5xFAD, amyloid β, Morris water maze

## Abstract

**Background**: Current treatment modalities for Alzheimer’s disease (AD), which is characterized by the accumulation of amyloid β (Aβ), have limitations with regard to their efficacy and safety, posing significant challenges for advances in healthcare. However, recent studies indicated that AD can be treated using monocyte-derived macrophages (MDMs). Reportedly, the protein CD300c regulates monocyte differentiation, indicating that targeting CD300c could offer a treatment for AD. **Methods**: To confirm this, we developed CB201, a fully human anti-CD300c antibody, and demonstrated its strong and specific binding to CD300c using surface plasmon resonance and binding ELISAs. **Results**: Treatment of THP-1 and human peripheral blood mononuclear cells with CB201 led to increased levels of pro-inflammatory cytokines and the differentiation of macrophages to MDMs. Moreover, the CB201-differentiated macrophages expressed cytokines and chemokines in a pattern that alleviates AD symptoms. In a 5xFAD mouse model, CB201 treatment improved memory and behavior in both the early and late stages of AD and reduced cerebral Aβ plaque load. **Conclusions**: These results indicate that CB201 promotes the differentiation of macrophages to MDMs and modulates AD-related inflammatory responses, thereby ameliorating the pathological features of AD. These findings identify CD300c as a potential therapeutic target for AD and indicate that CB201 is a promising candidate for its treatment.

## 1. Background

Although traditional drugs for treating patients with Alzheimer’s disease (AD), which is characterized by the accumulation of amyloid β (Aβ) [1,2], temporarily attenuate the symptoms, they do not fundamentally treat AD [3]. Aducanumab and Lecanemab have recently received accelerated approval from the Food and Drug Administration for the treatment of AD. Reportedly, these drugs induce the clearance of the brain Aβ plaque load, although concerns regarding their efficacy and potential side effects remain [4,5,6].

The established roles of monocyte-derived macrophages (MDMs) differentiated from peripheral blood monocytes [7] led to the emergence of strategies for treating AD. Reportedly, these macrophages can prevent or eliminate amyloid deposition in mouse models [8,9,10]. Given its utility in assessing the effects of potential AD treatments on intraneuronal Aβ42-induced neurodegeneration and amyloid plaque formation, the 5xFAD mouse model is widely used to study this disease [11,12].

The human CD300 multigene family comprises seven members located on chromosome 17 [13], which encode type I transmembrane proteins with a single IgV-like extracellular domain containing two disulfide bonds. Among these, CD300c is associated with adaptor proteins, such as the DNAX-associated proteins (DAP12 and DAP10), and the Fc receptor (FcR)γ chain [14]. CD300c is an activating receptor expressed in human monocytes, and in a previous study, we demonstrated that the engagement of CD300c by anti-CD300c antibodies activates MAPK and NF-κB and promotes macrophage differentiation [15]. On the basis of these findings, we hypothesized that, by promoting macrophage differentiation, an antibody targeting CD300c would reduce Aβ plaque formation. To test this hypothesis, we developed CB201, a fully human anti-CD300c antibody, screened using a synthetic human phage library, and found that monocytes that differentiate to macrophages via CB201 induction can clear Aβ from the brain, and accordingly, administered CB201 to 5xFAD model mice to assess its efficacy in treating AD.

## 2. Methods

### 2.1. Screening and Characterization of Monoclonal Antibodies

The affinity of CB201(CL7) to human CD300c immobilized on CM5 sensor chips was determined based on surface plasmon resonance (SPR) analysis. The SPR absorption of the samples was determined at 450 nm using a Biacore T200 system (Cytiva, Marlborough, MA, USA) and dedicated software (Biacore T200 software v3.2, Cytiva), and the dissociation constants (KD) were calculated as the dissociation rate (K_off_)/association rate (K_on_) with different concentrations of CB201 (0–6.25 µg/mL). In the binding enzyme-linked immunosorbent assay (ELISA), following coating with human CD300c recombinant protein, plates were treated with different concentrations of CB201 (2.5–10 µg/mL), washed, and subsequently incubated with horseradish peroxidase (HRP)-conjugated anti-human IgG (Sigma-Aldrich, St. Louis, MI, USA). The EC_50_ values were calculated using SoftMax^®^ pro software v7.1 (Molecular Devices, San Jose, CA, USA).

The binding of CB201 to human CD300c overexpressed on 293T cells (ATCC, Manassas, VA, USA) was determined using fluorescence-activated cell sorter (FACS) analysis. Cells were incubated in RPMI 1640 medium (Welgene, Gyeongsan-si, Gyeongsangbuk-do, Republic of Korea) containing 10% serum (Gibco, Waltham, MA, USA). Untransfected and hCD300c-overexpressing 293T cells were incubated with CB201, washed, and subsequently incubated with fluorescein isothiocyanate-conjugated anti-hIgG. The fluorescence intensity was measured using a CytoFLEX flow cytometer (Beckman Coulter, Brea, CA, USA) and dedicated software.

### 2.2. Macrophage Differentiation

Primary monocytes were isolated from human peripheral blood mononuclear cells (iXCells, San Diego, CA, USA) using a PAN monocyte isolation kit (Miltenyi Biotec, Bergisch Gladbach, Germany). For the multicytokine assays (#HCYTA-60 K-PX48, Sigma-Aldrich), primary monocytes were incubated with 10 μg/mL IgG or CB201 for 48 h, and the concentrations of cytokines/chemokines were measured using Luminex. THP-1 and PBMCs were treated with 10 μg/mL IgG (Invitrogen, Carlsbad, CA, USA), 10 μg/mL CB201, or 100 ng/mL lipopolysaccharide in RPMI 1640 medium without serum. The cell morphology was examined after treatment for 24 h, and the concentrations of tumor necrosis factor (TNF)-α, interleukin (IL)-1β, and IL-8 were measured using Quantikine kits (R&D Systems, Minneapolis, MN, USA), with the results being compared using ratio-paired *t*-tests (* *p* < 0.05, ** *p* < 0.01).

### 2.3. Scheme of Administration Using the 5xFAD Model

B6SJLF1/J male mice were purchased from the Jackson Laboratory(Bar Harbor, ME, USA). All animal experiments were approved by the KNOTUS Institutional Animal Care and Use Committee (approval numbers: 21-KE-206, 23-KE-0133). For the early-stage experiments, 6-month-old 5xFAD mice (*n* = 4 per group) were intraperitoneally administered with 200 μL of phosphate-buffered saline (PBS) or CB201, and wild-type B6SJLF1/J mice (*n* = 3) were administered PBS twice on days 0 (40 mg/kg) and 25 (10 mg/kg). For the late-stage experiments, 8.5-month-old 5xFAD mice (*n* = 4) were intraperitoneally administered PBS, CB201, or anti-mouse PD-L1 antibody (BioXCell, Lebanon, NH, USA), and wild-type B6SJLF1/J mice (*n* = 4) were administered PBS on days 0 and 3 at a dose of 10 mg/kg.

### 2.4. Multiplex Cytokine/Chemokines ELISA Kit Assay

The samples prepared as Section 2.2 were analyzed using Human Cytokine/Chemokine/Growth Factor Panel A from Millipore Sigma (Burlington, MA, USA). A total of 10 cytokines/chemokines were run for each sample: G-CSF, IL-17 A/F, CCL5, RANTES, MIP, IL-1Ra, IFN-γ, MIP-1α, IFN-γ, GM-CSF. The cytokine/chemokine levels were measured using a Luminex analyzer (Luminex, Austin, TX, USA). Sample measurements were conducted in accordance with the manufacturer’s protocol.f

### 2.5. Behavior Test

The Y-maze tests for the early- and late-stage groups were performed on day 28 after the administration started. The mice of each group were positioned at the end of the start arm and given 7 min to explore the maze freely. The frequency of arm entries and the right consecutive alternations were measured to calculate spontaneous alteration (% spontaneous alteration = total alteration number/(total entry number − 2) *×* 100). The Morris water maze tests were performed in the early-stage groups from day 35 to day 40 and in the late-stage groups from day 23 to day 28 after the administration started. The experimental setup consisted of a stainless-steel circular tank (diameter 150 cm, height 45 cm) divided into quadrants, a platform (diameter 10 cm, height 30 cm), and four visual cues attached to the walls of the tank. The tank was filled with water at approximately 22 ± 2 °C with white water-based paint. Cued learning was performed with the platform positioned 1 cm above the water’s surface on the first day and with the hidden platform from the second to the fifth day. On the sixth day, a prospective, randomized, open-blinded end-point (probe) trial was conducted. The platform was removed, and each mouse was positioned in the quadrant most distant from the previous platform location used during the second to fifth days. The maximum cut-off time per trial was 60 s. The escape latency (the time to reach the platform) and the path length (the distance to reach the platform) were measured and recorded. The swimming speed was determined by dividing the path length by the escape latency.

### 2.6. Immunohistochemistry

On Day 71 after the start of the administration, the mice were anesthetized, perfused with saline, and the brains were collected. The brains were fixed with 4% paraformaldehyde and paraffin-embedded. Immunohistochemistry staining was performed using anti-amyloid β Monoclonal Antibody (SIG-39320, Covance, Princeton, NJ, USA) and HRP-conjugated anti-mouse antibodies (K4000, DAKO, Santa Clara, CA, USA). The stained areas were measured on 5 coronal sections per mouse. The experimenters were blinded to the information about the mice during the histological analyses.

## 3. Results

### 3.1. Binding Affinity and Specificity of CB201 Against CD300c

**Figure 1:**
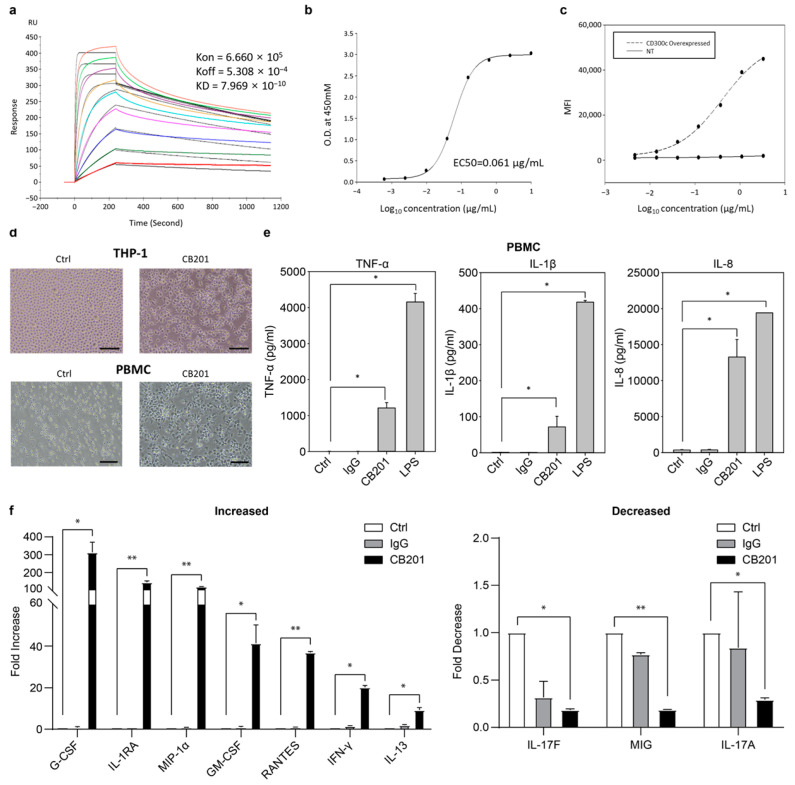
CB201 binding to the CD300c antigen and its effects on macrophage differentiation in vitro. (**a**) Binding affinity of CB201 to recombinant human CD300c antigen, as determined by SPR. The colored lines represent sensorgrams of different concentrations of the analyte used in the SPR analysis. (**b**) Determination of the EC_50_ of CB201 using binding ELISA. EC_50_ values are displayed on the graph. (**c**) Binding affinity of CB201 for exogenous CD300c, as determined by FACS. (**d**) Morphological differentiation of THP-1 and primary monocytes to MDMs induced by CB201. Magnification ×200, scale bar = 100 μm. (**e**) Induction of MDMs from primary monocytes upon treatment with CB201, confirmed using quantikine-ELISA. (**f**) Changes in cytokine/chemokine production in primary monocytes following CB201 treatment determined by multicytokine. Upper and lower parts show increases and reductions in secretion induced by CB201 treatment, respectively. Statistical significance was determined using ratio-paired *t*-tests; * *p* < 0.05, ** *p* < 0.01.

The binding affinity of CB201 to human CD300c was characterized based on SPR and binding-ELISA analyses. The assays showed a binding affinity of 0.8 nM and an EC_50_ of 0.061 nM (Figure 1a,b), thereby indicating that CB201 has a high binding affinity to human CD300c. To evaluate whether CB201 binds to CD300c on the surfaces of live cells, we analyzed the binding of CB201 to human CD300c overexpressed in 293T cells using flow cytometry and confirmed that CB201 bound specifically to native CD300c on the cell surface (Figure 1c).

### 3.2. Effects of CB201 on Macrophage Differentiation

To assess the functional effects of CB201 on immune cells, the primary monocytes and THP-1 cells were treated with CB201. Light microscopy revealed morphological changes in CB201-treated THP-1 and primary monocytes with good attachment (Figure 1d), along with approximately 12-, 8-, and 15-fold increases in the expressions of pro-inflammatory cytokines, such as TNF-α, IL-1β, and IL-8, respectively (Figure 1e).

To verify whether CB201 treatment can induce macrophage differentiation with MDM characteristics, we examined changes in the expression of AD-associated cytokines/chemokines. CB201 treatment promoted 313-, 144-, 120-, 41.3-, 36.9-, 20.2-, and 9.21-fold increases in the expressions of G-CSF, IL-1RA, MIP-1α/CCL3, GM-CSF, RANTES/CCL5, IFN-γ, and IL-13, respectively, and 0.19-, 0.19-, and 0.29-fold reductions in the expressions of IL-17F, IFN-γ (MIG)/CXCL9-induced monokines, and IL-17A, respectively (Figure 1f).

### 3.3. Behavioral Improvement in 5xFAD Mice

**Figure 2:**
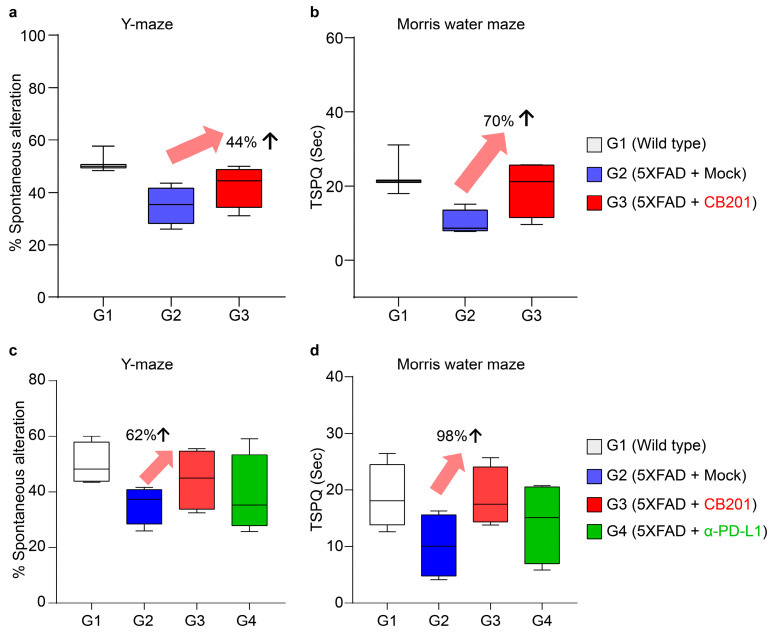
Behavioral changes induced by CB201 in Y-maze and on Morris water maze (MWM) tests. Alternation rate in the Y-maze test (**a**) and time spent in the target quadrant in the MWM test (**b**) in the early-stage groups (G1, *n* = 3; G2, *n* = 4; G3, *n* = 4). Alternation rate in the Y-maze test (**c**) and time spent in the target quadrant in the MWM test (**d**) in the late-stage groups (G1, *n* = 3; G2, *n* = 4; G3, *n* = 4; G4, *n* = 4).

Given the potential applicability of CB201-differentiated macrophages in the treatment of AD, we examined whether CB201 administration could alleviate AD symptoms in 5xFAD model mice. At the early stage, the administration of CB201 increased spontaneous alterations in the Y-maze by 44% and the time spent at the platform site in the Morris water maze (MWM) by 70% (Figure 2a,b). Similarly, in the late stage, CB201 improved performance in the Y-maze and on MWM tests by 62% and 98%, respectively, thereby revealing improvements in spatial learning and memory.

The therapeutic effects of PD-L1 blockade in AD using members of the B7 family, such as CD300c, have been well demonstrated [11,16]. Therefore, an anti-PD-L1 antibody was administered to AD mice to compare its efficacy with that of CB201. In both behavioral tests, the behavioral improvement in late-stage mice administered CB201 was greater than that induced by anti-PD-L1 Ab (α-PD-L1) (Figure 2c,d).

### 3.4. Reduction in Aβ Deposition

**Figure 3:**
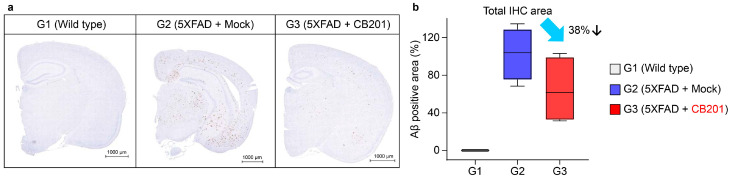
Reduction in amyloid β deposition by CB201 in the brains of 5xFAD mice. (**a**) Brain sections in the early-stage group with immunostaining of amyloid β peptides (scale bar = 1000 μm). (**b**) Quantification of amyloid β peptides (5 brain sections per group).

Given the behavioral improvement in AD mice administered CB201, we measured the levels of Aβ, a known cause of AD, in the brains of mice following CB201 administration. In line with expectations, CB201-administered mice showed a 38% reduction in the amyloid plaque burden throughout the brain, including the hippocampus, which is consistent with the behavioral improvements (Figure 3).

## 4. Discussion

In this study, we generated CB201 antibodies, which are fully human anti-CD300c monoclonal antibodies that bind specifically to native and recombinant CD300c proteins, and determined their characteristics and efficacy using SPR, binding-ELISA, and FACS physicochemical analyses.

The SPR and binding-ELISA revealed that CB201 bound specifically to the recombinant CD300c antigen at the sub-nanomolar level with high binding efficiency. Furthermore, a FACS analysis indicated that CB201 bound specifically to the native form of CD300c.

CB201 treatment also induced macrophage-like morphologies and promoted the expression of the pro-inflammatory cytokines TNF-α, IL-1β, and IL-8 in THP-1 and human primary monocytes. Furthermore, in mice, CB201 administration promoted MDM, activated MDM-promoted signaling, and increased the levels of inflammatory cytokines. Reportedly, an increase in MDMs ameliorates the symptoms of neurodegenerative diseases, thereby indicating the therapeutic potential of CB201 [9,17].

Additionally, different cytokines/chemokines associated with the alleviation of AD symptoms were secreted by macrophages differentiated in response to CB201 treatment. Elevated levels of G-CSF, GM-CSF, and IL-13, which have therapeutic effects on AD, and IL-1RA, MIP-1α, RANTES, and IFN-γ, which are anticipated to improve AD symptoms, were observed following CB201 administration. Conversely, we observed reductions in the levels of IL-17A, IL-17F, and MIG (detected at high levels in patients with AD), which are predicted to be associated with an alleviation of AD symptoms [16,18,19,20,21,22,23,24]. These findings imply that macrophages induced by CB201 treatment may make a positive contribution to alleviating AD symptoms.

We also found that CB201 administration enhanced the behavior of 5xFAD and APP/PS1 (an AD model induced by deposition of Aβ and pTau;) model mice. The efficacy of CB201 administration was determined using the Y-maze test for short-term memory improvement and the Morris water maze test for improvements in long-term memory and spatial learning. CB201 administration was found to enhance cognitive function in both the early and late stages, indicating that it can influence the overall progression of AD, as efficacy in the early and late stages implies the prevention and treatment of AD, respectively. Moreover, CB201 showed higher efficacy than that of the immunotherapeutic anti-PD-L1 antibody, which has been determined to improve AD symptoms via MDMs [9,17].

The use of MDMs is an emerging concept in AD treatment. Notably, MDMs replace exhausted microglia in the brain to remove the aggregates that induce neuronal damage. Consistent with the results of behavioral experiments, we observed reductions in Aβ aggregates throughout the brain following CB201 administration. However, further validation is required to confirm whether an improvement in AD symptoms following CB201 administration is induced by MDMs. To verify whether the aggregates were reduced by MDMs, we transplanted GFP-labeled bone marrow cells into APP/PS1 model mice.

Given that PD-1/PD-L1 plays a specific role in regulating T cells, and that CD300c, the target of CB201, specializes in regulating monocytes and macrophages, the respective combination therapy could potentially exert a synergistic effect in the improvement of AD symptoms. Additional experiments are currently underway to investigate the therapeutic efficacy of this approach.

## 5. Conclusions

This study is the first to demonstrate the potential of CD300c, a protein involved in the regulation of monocyte differentiation, in AD treatment and that the administration of a humanized anti-CD300c antibody, CB201, improves behavior and reduces Aβ deposition in AD mice.

## Data Availability

The datasets used and/or analyzed during the current study are available from the corresponding author upon reasonable request.

## Abbreviations

AD	Alzheimer’s disease
Aβ	amyloid β
ELISA	enzyme-linked immunosorbent assay
FACS	Fluorescence-activated cell sorter
FcR	Fc receptor
HRP	horseradish peroxidase
IL	interleukin
KD	dissociation constants
K_off_	dissociation rate
K_on_	association rate
MDMs	monocyte-derived macrophages
MIG	expression of IL-17F, IFN-γ
MWM	Morris water maze
SPR	surface plasmon resonance
TNF	tumor necrosis factor
